# Youth not engaged in education, employment, or training: a discrete choice experiment of service preferences in Canada

**DOI:** 10.1186/s12889-024-18877-0

**Published:** 2024-05-27

**Authors:** Meaghen Quinlan-Davidson, Mahalia Dixon, Gina Chinnery, Lisa D. Hawke, Srividya Iyer, Katherine Moxness, Matthew Prebeg, Lehana Thabane, J. L. Henderson

**Affiliations:** 1 Centre for Addiction and Mental Health, 80 Workman Way, Toronto, ON M6J 1H4 Canada; 2 Dalla Lana School of Public Health, University of Toronto, Toronto, ON Canada; 3 Orygen, Parkville, VIC Australia; 4 Department of Psychiatry, University of Toronto, Toronto, ON Canada; 5 Department of Psychiatry, McGill University, Montreal, QC Canada; 6 Douglas Research Centre, Montreal, QC Canada; 7 Batshaw Youth and Family Centres, Montreal, QC Canada; 8 Department of Psychiatry, The Hospital for Sick Children, Toronto, ON Canada; 9 Department of Health Research Methods, Evidence, and Impact, McMaster University, Hamilton, ON Canada; 10 St Joseph’s Healthcare Hamilton, Hamilton, ON Canada; 11 Faculty of Health Sciences, University of Johannesburg, Johannesburg, South Africa

**Keywords:** Youth mental health and substance use, Youth not in education, Employment, Or training, Service preferences, Discrete choice experiment

## Abstract

**Background:**

Prior research has showed the importance of providing integrated support services to prevent and reduce youth not in education, employment, or training (NEET) related challenges. There is limited evidence on NEET youth’s perspectives and preferences for employment, education, and training services. The objective of this study was to identify employment, education and training service preferences of NEET youth. We acknowledge the deficit-based lens associated with the term NEET and use ‘upcoming youth’ to refer to this population group.

**Methods:**

Canadian youth (14–29 years) who reported Upcoming status or at-risk of Upcoming status were recruited to the study. We used a discrete choice experiment (DCE) survey, which included ten attributes with three levels each indicating service characteristics. Sawtooth software was used to design and administer the DCE. Participants also provided demographic information and completed the Global Appraisal of Individual Needs–Short Screener. We analyzed the data using hierarchical Bayesian methods to determine service attribute importance and latent class analyses to identify groups of participants with similar service preferences.

**Results:**

A total of *n*=503 youth participated in the study. 51% of participants were 24–29 years of age; 18.7% identified as having Upcoming status; 41.1% were from rural areas; and 36.0% of youth stated that they met basic needs with a little left. Participants strongly preferred services that promoted life skills, mentorship, basic income, and securing a work or educational placement. Three latent classes were identified and included: (i) job and educational services (38.9%), or services that include career counseling and securing a work or educational placement; (ii) mental health and wellness services (34.9%), or services that offer support for mental health and wellness in the workplace and free mental health and substance use services; and (iii) holistic skills building services (26.1%), or services that endorsed skills for school and job success, and life skills.

**Conclusions:**

This study identified employment, education, and training service preferences among Upcoming youth. The findings indicate a need to create a service model that supports holistic skills building, mental health and wellness, and long-term school and job opportunities.

**Supplementary Information:**

The online version contains supplementary material available at 10.1186/s12889-024-18877-0.

## Background

Youth not in education, employment, or training (NEET) struggle to navigate school to work transitions and experience difficulties accessing jobs [1]. These youth are disconnected from school, have limited work experience [2], and experience a loss of economic, social, and human capital [3]. NEET status is associated with lower education, parental unemployment, low socioeconomic status, low self-confidence, more precarious housing, and young parenthood [4–8]. In Canada, the percentage of NEET youth (15–29 years) was estimated at 11% in 2022 [9]. Importantly, NEET status is not homogenous across the country, ranging from 36% in Nunavut, 20% in Northwest Territories, and 17% in Newfoundland and Labrador to 10% in Quebec, Prince Edward Island and British Columbia) [10]. Supporting and protecting these marginalized youth remains a challenge, particularly in light of the Coronavirus disease 2019 (COVID-19) pandemic, which adversely impacted the school to workforce transition for youth across the country [11]. Although the term NEET has been used to describe this population, it is considered stigmatizing and associated with a deficit-based lens[12]. As such, and in consultation with one of our youth team members, we refer to this population as ‘Upcoming youth’[13].

Upcoming status has gained attention across Canada in recent decades [14]. As an illustration of this focus, federal, provincial/territorial, and local programs exist to support Upcoming youth across the country [15]. Despite these efforts, evidence indicates program fragmentation, limited coordination across sectors and regions, and a lack of evaluation of these programs [16]. Further, these programs may be available to youth on a short-term basis and specific to youth who meet education, income, and age criteria [17]. There is a lack of knowledge of how to (re)engage Upcoming youth in general education and employment support services. Often the same limited outcomes are measured and reported (e.g., job attainment) with services focusing on these outcomes. At the same time, youth have not been asked what outcomes they prefer and accordingly what services they would like. Indeed, selective outcome reporting and lack of engagement of youth impairs the quality of evidence and contributes to research waste [18]. Given the heterogeneity of Upcoming status, this lack of evidence is particularly important for subgroups of youth (e.g., geographic location; socioeconomic status; mental health status) who face challenges in the school-to-work transition.

Prior global research has emphasized the importance of integrated, coordinated interventions that offer a range of support services (e.g., on-the-job, classroom-based, and social skills training) to prevent and reduce Upcoming status [19–23] [24]. Integrated youth service (IYS) models, which integrate education, employment, mental and physical health, substance use, peer support, and navigation in one, youth-friendly location have been established in Canada [25]. IYS deliver services that meet the needs, goals, and preferences of youth, and hold promise in serving vulnerable Upcoming youth through the provision of holistic services in a youth-friendly environment. Indeed, IYS models are investigating how to optimize employment, education, and training services as a critical component of supporting youth wellbeing and their successful transition to adulthood. This point is particularly important as Upcoming youth experience greater mental health and substance use (MHSU) concerns compared to youth who do not identify as Upcoming [26, 27].

An essential component to designing and enhancing health and social services for Upcoming youth is understanding their perspectives [28]. Yet, there is a lack of evidence on Upcoming youth’s perspectives and preferences for employment, education, and training services within the Canadian context. For interventions to be relevant to the needs and experiences of youth—which will increase their chances of using the services and benefiting from them—it is important to understand what youth aim to achieve when participating in an intervention. Engaging youth in identifying service components and interventions will ensure that programs and services are relevant, feasible, and appropriate to this population group [29].

An approach that can be used to identify the demands and preferences of youth is the discrete choice experiment (DCE) [30, 31]. The DCE is a quantitative method that requires participants to state their choice over sets of alternatives described in terms of several characteristics called attributes and the value placed on each attribute [30, 31]. In this way, the DCE is able to identify the importance of attributes along which a variety of service options vary, as well as service preferences among subgroups. DCEs are one of the most popular methods for eliciting stated- preferences in health care [32, 33]. They force participants to make trade-offs, identifying the importance of different service attributes [32, 33]. Previous findings generated from DCE studies have been useful in informing service design and delivery, resource allocation, and policies, including the preferred design of IYS services [34–37].

Understanding service preferences from the perspective of Upcoming youth is critical for the development of interventions and policies that will help youth navigate the school-to-work transition. As such, the objective of the current study was to identify employment, education and training service preferences of Upcoming youth. As our approach to COVID-19-related impacts shifts, the need for this research is more urgent than ever, as a way to support vulnerable youth, reduce Upcoming status, prevent further exclusion, and help them on their path towards adulthood.

## Methods

### Discrete Choice Experiment (DCE)

A discrete choice experiment (DCE) methodology was used in this study, as described in the study protocol [13]. We followed the International Society for Pharmacoeconomics and Outcomes Research (ISPOR) guidelines on good research practices for conjoint analysis [38]. Attributes and levels were developed using the following methods. First, we reviewed the literature on relevant and preferred services for youth with Upcoming and at-risk Upcoming status [26]. An initial set of six attributes with three to four levels was developed from the literature review, highlighting components such as mental health, goals, and skills training. Second, focus groups were conducted among youth (16–29 years) with Upcoming and at-risk Upcoming status across Canada to obtain youth feedback on proposed service outcomes [39]. Thematic analysis [40] of the focus group data identified prominent attributes and levels, including skills training, mentorship, and networking. The project team included youth team members with lived/living experience of MHSU concerns and researchers; meetings were held with the team to refine the attributes and levels.

The list of attributes and levels were piloted among *n *= 9 youth (16–29 years) across Canada. Pilot participants completed the DCE with a member of the project team. The aim of the pilot was to obtain youth feedback on the proposed list of attributes and levels, as well as the design and functionality of the DCE. Based on pilot feedback, a final list of attributes and levels was developed. The final DCE list included ten attributes, each with three levels. The attributes included mentorship; skills for school and job success; technical skills; life skills; basic income; networking opportunities; securing a work or educational placement; career counselling; access to free mental health and substance use services; and support for mental health and wellness in the workplace. Using a 3 × 3 partial-profile design, we used Sawtooth software (version 9.14.2) [41] to administer the 14 randomized choice tasks. This design was chosen to optimize orthogonality, minimize participant burden, and ensure data robustness [42]. Table 1 shows a sample choice task; Additional File 1 contains the full list of attributes and levels in the study.

**Table 1:** Sample of discrete choice experiment choice task

*If you were seeking employment, education or training services, what would you want to get out of these services?*			
	**Option 1**	**Option 2**	**Option 3**
**Securing a Work or Educational Placement**	Support to secure long-term job positions or educational placements that align with career interests and long-term goals	Support to find temporary job positions or educational placements to explore career options and fit	Support to secure any job position or educational placement, without considering longer-term career interests or paths
**Career Counselling**	Do not provide me with career counselling	Provide career counselling to help me figure out my career goals	Provide career counselling to help me figure out my career goals and how to create a resume/CV
**Skills for School and Job Success**	Free training that includes concrete skills like time management AND problem solving	Free training that includes concrete skills like time management and problem solving AND soft skills like professionalism, communication and relationship building	Free training focused on concrete skills like time management

### Participants and procedure

The study was approved by the Centre for Addiction and Mental Health’s (CAMH) Research Ethics Board in Toronto, Canada. This study consisted of *n* = 503 youth (14–29 years), recruited over a three-month period in late 2022 and early 2023. The sample size was based on a priori power calculations and exceeds the sample size of most DCE studies [13]. Study flyers with survey links were distributed through internal CAMH and external professional networks, as well as through social media (Facebook and Instagram).

Participants were eligible to complete the DCE if they were between the ages of 14 and 29 years; lived in Canada at the time of survey completion; and identified as Upcoming status or having ever been concerned of being at-risk of Upcoming status (self-identified). They were screened through an online survey sent via email, hosted on REDCap electronic software [43]. Participants gave informed consent and filled out anti-spam and eligibility questions. Those who were eligible were sent a link to complete the DCE through Sawtooth Software [41]. The survey was in English only. They also filled out self-report questionnaires on demographics, and mental health and substance use. Reminder emails to complete the survey were sent to participants once per week, with a maximum of three reminders sent. A total of *n* = 515 participants initiated the survey and *n* = 503 completed the survey, yielding a response rate of 97.7%. The median time to complete the DCE was 20.63 min. Participants received a $30 gift card as honorarium for survey completion.

### Measures

#### Mental health and substance use measures

Participants completed the Global Appraisal of Individual Needs–Short Screener (GAIN-SS) (version 3) [44]. Internalizing disorders (depression, anxiety, somatic complaints, trauma etc.); externalizing disorders (hyperactivity, conduct problems, attention deficits, impulsivity etc.); and substance use disorders are domain subscales that are screened in the GAIN-SS [44]. The GAIN-SS also includes a crime/violence domain, however, low level of endorsement in this study precluded the inclusion of this subscale. Participants rated each administered symptom “never” to “within the past month”, indicating how recently they experienced symptom difficulties. Within each domain subscale, endorsed past month symptoms were counted and summed. Scores could range between 0–6, 0–7, and 0–5 for the Internalizing, Externalizing, and Substance Use Problems domain, respectively. Following previous literature, three or more items endorsed within the past month indicate a high likelihood of needing services and/or meeting threshold criteria for psychiatric diagnoses [44, 45].

#### Covariates

Demographic characteristics were collected. We included age (categorical measure), gender identity (man/boy [cis, trans]; woman/girl [cis, trans]; Gender diverse); ethnicity (White; Indigenous, Black, Asian, Mixed); region in Canada (Prairies, Western/Northern, Atlantic, Central); self-rated physical and mental health (good/very good/excellent; fair/poor) [46]; socioeconomic status (live comfortably; income meets needs with a little left; just meet basic expenses; don’t meet basic expenses); living arrangement (alone; with partner; with family; other); and area of residence (large city and suburbs of large city; small city, town, village or rural area).

#### Youth engagement

Following the McCain Model of Youth Engagement [47], and working with the Youth Engagement Initiative at the Margaret and Wallace McCain Centre for Child, Youth & Family Mental Health, we engaged youth throughout the study. To enhance study design, promote youth buy-in, and relevance of the study, youth were involved from project inception and implementation of study activities to interpretation of findings and manuscript development.

#### Statistical analysis

Statistical analyses were performed using Sawtooth Software version 9.14.2 [41] and Stata version 16.1 [48]. Descriptive statistics were calculated for all study variables overall and by latent-class grouping. Using hierarchical Bayesian methods within Sawtooth Software [41], utility estimates were calculated for each participant. Standardized zero-centered utilities were used and the average utility range of attribute levels was set to 100 (49) to calculate the estimates. Attributes with higher utility estimates indicated higher relative value compared with other attributes (Table 2).

**Table 2:** Overall average attribute importance scores and standard errors

	I	SE
Life Skills	13.58	0.23
Mentorship	12.52	0.23
Basic Income	11.84	0.21
Securing a Work or Educational Placement	11.73	0.20
Skills for School and Job Success	10.68	0.16
Support for Mental Health and Wellness in the Workplace	10.32	0.21
Networking Opportunities	8.69	0.13
Access to Free Mental Health and Substance Use Services	8.02	0.19
Career Counselling	7.47	0.14
Technical Skills	5.15	0.12

*I* Importance scores, *SE* Standard errors

To identify groups of participants with similar service preferences, we conducted latent class analyses [41]. To belong to a latent class, probabilities were assigned to each participant. Using different starting seeds, five replications for each latent class group was calculated, with log-likelihood decreases of 0.01 or less indicating convergence. Based on the analysis, we retained a three-class model. This model was determined by analyzing the Bayesian Information Criteria (BIC), Akaike Information Criteria (AIC), Consistent Akaike Information Criterion (CAIC), and Akaike’s Bayesian Information Criterion (ABIC); latent class sizes; and the interpretation of latent class groupings (Table 3.). Team discussions with youth team members were held to review the importance scores and rankings and establish the names of the latent glass groupings (Table 4). Stata 16.1 [48] was used to compare latent classes on demographic characteristics and GAIN-SS scores using chi-square tests.

**Table 3:** Fit indices for latent class solutions ranging from one to five classes

Number of Classes	Log-likelihood	Percent Certainty	AIC	CAIC	BIC	ABIC	Chi-Sq value
1	-6762.75	12.59	13,565.51	13,722.70	13,702.70	13,639.14	1947.35
2	-6658.40	13.93	13,398.81	13,721.05	13,680.05	13,549.76	2156.05
3	-6573.75	15.03	13,271.50	13,758.80	13,696.80	13,499.78	2325.36
4	-6499.03	15.99	13,164.06	13,816.41	13,733.41	13,469.66	2474.79
5	-6446.51	16.67	13,101.02	13,918.42	13,814.42	13,483.93	2579.84

*AIC* Akaike information criterion, *CAIC* Consistent akaike information criterion, *BIC* Bayesian information criterion, *ABIC* Akaike’s bayesian information criterion

**Table 4:** Attribute importance scores and rankings by latent class

	Focused Job and Educational Services (*n* = 204)		Mental Health and Wellness (*n* = 171)		Holistic Skills Building (*n* = 128)	
	I	R	I	R	I	R
Mentorship	12.23	3	12.64	1	12.83	2
Skills for School and Job Success	10.58	5	9.60	6	12.29	3
Technical Skills	4.68	10	6.33	10	4.33	10
Life Skills	13.36	1	11.88	3	16.22	1
Basic Income	11.52	4	12.29	2	11.76	4
Networking Opportunities	8.50	7	9.32	8	8.15	7
Securing a Work or Educational Placement	13.29	2	9.93	5	11.65	5
Career Counselling	7.98	8	7.45	9	6.68	8
Access to Free Mental Health and Substance Use Services	7.81	9	9.37	7	6.53	9
Support for Mental Health and Wellness in the Workplace	10.05	6	11.19	4	9.57	6

*I* Importance Scores, *R* Rankings

## Results

Table 5 presents participant demographic characteristics. The majority of participants were between 24 and 29 years of age; lived in urban areas; were engaged in employment and training only; and identified as White and girl/woman (Trans, cis). Almost two thirds of participants (65.79%) met threshold criteria for an internalizing disorder, followed by 36.62% with an externalizing disorder, and 8.65% with a substance use disorder.

**Table 5:** Demographic characteristics  (*n*=503)

	*n* (%)
*Age (years)*	
14–20	126 (25.30)
21–23	118 (23.69)
24–29	254 (51.00)
*Race/Ethnicity*	
Indigenous	26 (5.17)
Asian	85 (17.07)
Latin American and Caribbean	9 (1.79)
White	276 (54.87)
Black	23 (4.57)
Middle Eastern	5 (0.99)
Mixed	21 (4.17)
Multiple races selected	58 (11.53)
*Gender Identity*	
Boy/Man (trans, cis)	90 (17.89)
Girl/Woman (trans, cis)	268 (53.28)
Gender Diverse	145 (28.83)
*School* + *Employment Status*	
Student only	67 (13.48)
Student & Employed, Training	153 (30.78)
Upcoming	93 (18.71)
Employed, Training only	184 (37.02)
*Living Arrangement*	
Alone	65 (12.92)
With partner	131 (26.04)
With family	205 (40.76)
Other	102 (20.28)
*Region*	
Prairies	137 (27.29)
Western/northern Canada	122 (24.30)
Atlantic	85 (16.93)
Central	158 (31.47)
*Born in Canada*	
Yes	408 (81.27)
No	94 (18.73)
*Language*	
English	426 (84.89)
Another language	77 (15.31)
*Urban/Rural*	
Large city + suburbs	296 (58.85)
Small city + town	207 (41.15)
*Socioeconomic Status*	
Live comfortably	93 (18.83)
Meet needs with a little left	178 (36.03)
Just meet basic expenses	147 (29.76)
Don’t meet basic expenses	76 (15.38)
*Physical Health*	
Good/Very good/Excellent	266 (53.09)
Fair/Poor	235 (46.91)
*Mental Health*	
Good/Very good/Excellent	132 (26.45)
Fair	181 (36.27)
Poor	186 (37.27)
Screened positive for Internalizing Disorder^a^	327 (65.79)
Screened positive for Externalizing Disorder^a^	182 (36.62)
Screened positive for Substance Use Disorder^a^	43 (8.65)

^a^Likelihood of meeting threshold of diagnostic criteria

### Overall service preferences

The overall service preferences and importance scores of participants are presented in Table 2. Participants positively endorsed services that promoted life skills, mentorship, basic income, and securing a work or educational placement. Participants were least likely to endorse technical skills. Within life skills services, youth positively endorsed services that included managing finances, taxes, and skills associated with self-care and cooking. The provision of a mentor who worked within the participant’s field of interest was preferred by all youth. Participants positively endorsed basic income until having secured employment that matched the basic income level. All youth preferred services that provided support to secure long-term job positions or school placements that aligned with their career interests or long-term goals.

Table 3. illustrates the fit indices of the latent class analysis. A three-class model was retained based on fit, size of latent class grouping, and interpretation of findings. Attribute importance scores and rankings are presented in Table 4 by latent class. There were some commonalities identified across the latent classes. All latent classes positively endorsed services that offered mentorship (mentors in their field of interest, with similar backgrounds, or peer mentors), basic income, and networking. Youth preferred the provision of a mentor with work experience in their field of interest. Participants also positively endorsed receiving basic income until 25 years of age (regardless of school or job status), or until they had found a job that matched the basic income level. In addition, all participants endorsed skills to network and opportunities to network in their area of interest.

Over 60% of participants from all of the latent classes reported fair/poor mental health. In addition, over 60% of participants in each latent class grouping met threshold criteria for an internalizing disorder, compared to the other GAIN-SS disorders. Furthermore, more participants identified as having lived in large cities/suburbs compared to small cities/towns in each latent class.

### Latent Class 1: Job and educational services

The first latent class endorsed services that focused on education and long-term job services, focusing on a career trajectory (*n* = 204, 38.9%). Attributes that drove these decisions included career counselling and securing a work or educational placement. Youth positively endorsed career counselling that helped to figure out career goals, create a resume, and complete job applications. Further, youth positively endorsed receiving long-term job positions or school placements that align with their career interests and long-term goals, as opposed to temporary or any job position. Participants from this latent class (Table 6) were more likely to be 24–29 years of age compared to other ages. Approximately 22.06% of youth in this latent class identified as Upcoming.

**Table 6:** Demographic and clinical characteristics by latent class (*n* = 503)

		**Job and Education Services (** ***n*** ** = 204)**	**Mental Health and Wellness Services (** ***n*** ** = 171)**	**Holistic Skills Building Services (** ***n*** ** = 128)**	**Chi-sq**	***p***	**Cram V**
	**Categories**	***n*** (%)	***n*** (%)	***n*** (%)			
Age (years)	14–17	14 (6.97)	25 (15.15)	17 (13.49)	9.18	0.06	0.10
	18–23	72 (35.82)	59 (35.76)	52 (41.27)			
	24–29	115 (57.21)	81 (49.09)	57 (45.24)			
Race/Ethnicity	White	118 (57.84)	83 (48.54)	75 (58.59)	4.21	0.12	0.09
	Indigenous, Black, Asian, Mixed	86 (42.16)	88 (51.46)	53 (41.41)			
Gender Identity	Boy/Man (trans, cis)	32 (15.69)	39 (22.81)	19 (14.84)	7.74	0.10	0.09
	Girl/Woman (trans, cis)	105 (51.47)	94 (54.97)	69 (53.91)			
	Gender Diverse	67 (32.84)	38 (22.22)	40 (31.25)			
School + Employment Status	Student only	25 (12.25)	27 (15.79)	16 (12.5)	5.82	0.44	0.08
	Student & employed	61 (29.90)	58 (33.92)	37 (28.91)			
	Upcoming	45 (22.06)	29 (16.96)	20 (15.63)			
	Employed only	73 (35.78)	57 (33.33)	55 (42.97)			
Living arrangement	Alone	24 (11.76)	26 (15.20)	15 (11.72)	4.07	0.67	0.06
	With partner	57 (27.94)	41 (23.98)	33 (25.78)			
	With family	76 (37.25)	74 (43.27)	55 (42.97)			
	Other	47 (23.04)	30 (17.54)	25 (19.53)			
Born in Canada	Yes	182 (89.22)	128 (74.85)	98 (77.17)	14.49	< 0.001	0.17
	No	22 (10.78)	43 (25.15)	29 (22.83)			
Region	Prairies	51 (25.00)	50 (29.24)	36 (28.35)	3.46	0.75	0.06
	Western/northern Canada	45 (22.06)	46 (26.90)	31 (24.41)			
	Atlantic	36 (17.65)	27 (15.79)	22 (17.32)			
	Central	72 (35.29)	48 (28.07)	38 (29.92)			
Language	English	182 (89.22)	135 (78.95)	109 (85.16)	7.59	0.02	0.12
	Another language	22 (10.78)	36 (21.05)	19 (14.84)			
Urban/Rural	Large city + suburbs	124 (60.78)	93 (54.39)	79 (61.72)	2.16	0.34	0.07
	Small city + town	80 (39.22)	78 (45.61)	49 (38.28)			
Socioeconomic Status	Live comfortably	36 (17.73)	40 (24.24)	17 (13.49)	8.19	0.22	0.09
	Meet needs with a little left	69 (33.99)	57 (34.55)	52 (41.27)			
	Just meet basic expenses	62 (30.54)	44 (26.67)	41 (32.54)			
	Don’t meet basic expenses	36 (17.73)	34 (14.55)	16 (12.70)			
Physical Health	Good/Very good/Excellent	102 [50]	102 (60.36)	62 (48.44)	5.48	0.06	0.10
	Fair/Poor	102 (50)	67 (39.64)	66 (51.56)			
Mental Health	Good/Very good/Excellent	45 (22.28)	57 (33.53)	30 (23.62)	6.71	0.03	0.12
	Fair/Poor	157 (77.72)	113 (66.47)	97 (76.38)			
Internalizing Disorder^a^	High Symptoms	141 (70.15)	102 (60.36)	84 (66.14)	3.92	0.14	0.09
Externalizing Disorder^a^	High Symptoms	72 (35.82)	58 (34.32)	52 (40.94)	1.46	0.48	0.05
Substance Use^a^	High Symptoms	20 (9.95)	15 (8.88)	8 (6.30)	1.33	0.51	0.05

^a^Past month symptoms

### Latent Class 2: Mental health and wellness services

The second latent class endorsed mental health and wellness services (*n* = 171, 34.9%). This latent class preferred that services offer support for mental health and wellness in the workplace and free mental health and substance use services. Specifically, participants positively endorsed the provision of on-site in-person, individual mental health and substance use services, as opposed to the provision of virtual or in-person group services. Further, youth positively endorsed ongoing access to a support worker to help in securing accommodations in the workplace, as opposed to learning how to advocate for oneself in workplace or support during job onboarding. Participants that endorsed this latent class (Table 5) tended to identify as Indigenous, Black, Asian, and Mixed; girl/woman (cis, trans); both student and employed; income met needs with a little left; and rated their physical health as good/excellent. Approximately 16.96% of youth in this latent class identified as Upcoming.

### Latent Class 3: Holistic skills building services

Skills building was the focus of the third latent class (*n *= 128, 26.1%). Participants positively endorsed skills for school and job success, as well as life skills. Specifically, youth were interested in learning about how to organize time; prioritize tasks; identify problems and solutions; as well as professionalism, communication and relationship building. Youth were also interested in life skills that focused on learning about how to manage finances and taxes, as well as self-care and cooking. Participants in this latent class (Table 5) tended to identify as White; employed only; living in an urban area; and income that just met basic expenses. Approximately 15.63% of youth in this latent class identified as Upcoming.

## Discussion

To our knowledge, this study was the first to identify employment, education, and training service preferences among Upcoming youth and those at-risk of Upcoming status using discrete choice experiment methods. The findings indicate that overall, youth value services that enhance their ability to deal effectively with life demands; receive advice and guidance by a mentor; and obtain financial support through basic income. In examining youth participants by latent class, the findings indicate a need to create a service model that supports long-term school and job opportunities, holistic skills building, and mental health and wellness. Job and educational services prioritized long-term job and school placements, with career counselling. Mental health and wellness services endorsed free, easily accessible and in-person support services. Meanwhile, holistic skills building focused on problem solving, communication, relationship building, and organization of time, as well as building skills to help youth manage daily life.

Participants highly endorsed services that promote life skills, mentorship, and basic income. For life skills, participants valued skills that included managing finances, taxes, and skills like self-care or cooking. Participants may have valued this service attribute because life skills empower youth. These skills are positive behaviours that give youth the knowledge, values, attitudes, and abilities necessary to effectively meet and deal with everyday challenges [50, 51]. Prior research has shown how these skills strengthen psychosocial competencies, promote health and social relationships, and protect against risk-taking behaviours [51].

For mentorship, participants valued having a mentor who has work experience in the field they are interested in. Prior research has shown the negative associations between unemployment, exclusion, and economic hardship among Upcoming youth [52, 53]. Participants may have chosen this service attribute as mentoring is a key component of career development. Career mentoring provides opportunities for career exploration and strengthening decision-making within this domain [54–57]. Research has shown the benefits of mentorship. Mentors are a positive resource, providing support and guiding youth as they navigate and succeed in their careers [54, 58, 59].

Youth also prioritized receiving basic income until they secured employment that matches the basic income level. Empirical evidence has shown associations between income and youth mental health outcomes [60–62]. Indeed, Johnson et al. [63] [63] posit that a universal basic income can positively affect health through behaviour, resources, and stress. Defined as income support to populations with minimal or no conditions [64], prior research has shown the benefits of a basic income plan in terms of poverty reduction, improvements in physical and mental health, economic growth, and human capital gains [65–69]. In a qualitative study in England [70], youth (14–24 years) reported that a universal basic income plan would improve their mental health through financial security, agency, greater equality, and improvements in relationships.

Differences in service preferences were observed among youth subgroups based on the identified latent classes. Youth who identify as Indigenous, Black, Asian, and Mixed prioritized mental health and wellness services compared to youth who identify as White. Previous literature has showed that Indigenous*, Black,* and racialized youth have experience longer wait times and poorer quality of mental health care compared to their White counterparts [71–73]. Prior literature has described how MHSU systems often do not consider or address the discrimination, systemic racism, economic marginalization, and intergenerational traumas that Indigenous, Black, and racialized populations experience within and outside of the service system [74, 75]. These negative experiences adversely affect their access to and quality of MHSU care, leading to inequities in MHSU outcomes. To ensure that mental health and substance use services are culturally responsive, safe, effective, and available to Indigenous, Black, and racialized youth, services should incorporate their perspectives into service design and delivery. The finding that youth 24–29 years of age endorsed job and educational services focused on long term career planning could be attributed to being more advanced in thinking about their careers and a desire to find a career as opposed to a job [57, 76, 77]. It could also be due to older youth experiencing poorer labour market conditions [19, 78, 79]. To improve long-term job opportunities for youth, Canada’s labour standards need to be updated, ensuring protection and benefits to informal and non-standard youth workers (17).

A critical component for education, employment, and training services is raising awareness about these services and their benefits among youth. A 2019 survey among NEET youth (16–29 years) in Canada showed that 54% reported a hard time finding information on the labour market services, while 42% said the information available on these services was not easy to understand [80]. One way to address this issue is by delivering services to Upcoming youth at the local, community level. In fact, as IYS strengthen education, employment and training services, these community-based services can support youth by connecting them with local job opportunities. IYS can also work with other public, private and community organizations to change local, fragmented school and work policies [19]. Another way to address this issue would be to provide access to this information at an earlier age, as shown in a parliamentary enquiry in Victoria, Australia in which career management was recommended for incorporation into primary school curriculum[81]. 

All three latent classes preferred services that provided mentorship, basic income, and networking opportunities. Youth value mentorship opportunities from individuals with experience in their field of interest. Similarly, youth prioritized networking opportunities in their field of interest. Federal, provincial/territorial and local programs could harness this preference by creating mentorship and networking structures across public, private, and community organizations for youth [17]. Further, the provision of basic income would help support youth as they re-engage with school and the labour market [17]. Interestingly, technical skills were not endorsed by youth in this study. Although technical skills are endorsed as part of technical and vocational education and training programs [82], it may be that youth were not as concerned about enhancing technical skills as they were other services. Future research should investigate youth experiences of technical skill programs.

It is important to note that participants in all latent classes endorsed poor mental health, while a higher proportion of youth screened positive for internalizing disorders compared to other disorders. These findings are in line with prior literature, particularly in light of the COVID-19 pandemic [83–91]. It could also reflect the positionality of the researchers. The survey was administered by CAMH, a mental health teaching hospital, and could have been seen more among youth connected with mental health services compared to those not connected to CAMH. Prior research has showed that life skills training can promote positive development, mental wellbeing, and prevent risky behaviours [92, 93]. The prioritization of long-term school and job placements among youth with mental health concerns indicates a need to strengthen these services for this cohort.

In fact, in 2020 the Individual Placement and Support (IPS) model [94, 95], which provides mental health service users with personalized vocational support alongside mental health support to obtain employment, education, and training opportunities was launched in Alberta, British Columbia, Nova Scotia, Ontario and Quebec to strengthen existing IYS, including ACCESS Open Minds, Foundry, and Youth Wellness Hubs Ontario [96]. The program was implemented in 12 hubs across the country and is currently being evaluated. Despite the challenges that have arisen over the course of the pandemic, COVID-19 has highlighted an opportunity to improve the education, employment and training support systems that serve these youth. Some of the core principles of implementation of the IPS model align with findings from the current study and include integration of mental health treatment teams, employment specialists to support young people as they navigate the labour market, rapid job search approaches, and tailored job supports, among other principles [94].

Indeed, in building on the services endorsed in this study, it would be important to incorporate an evaluation framework such as the Consolidated Framework for Implementation Research [97] to evaluate the effectiveness and impact of these services. Determining potential outcomes that could be measured would also be important. Following the IPS model, for job and educational placements, services could implement the Youth Employment and Education Survey [94, 95]. Potential outcomes could include status of school or employment, job permanency, educational placement duration, and satisfaction with the program, among others. For mental health and wellness support services, outcomes could focus on the number of in-person visits, satisfaction with the services, and self-reported mental health, among others. For holistic services, potential outcomes could focus on reporting and monitoring self-reported goals for problem-solving and communication, among others. It would be important to continuously assess and match services to Upcoming youth preferences.

We would like to acknowledge some limitations. This study includes a non-randomized sample of youth across Canada. Our study included less than 20% of youth who identified as Upcoming, which limits our ability to generalize the findings to this population group. Further research is needed among youth who identify as Upcoming to determine if these education, employment, and training services represent their preferences. Further, youth without stable and consistent internet access would also have been missed. We were unable to recruit large populations of youth from specific Indigenous and racialized backgrounds, although these did account for nearly half the sample. These groups may have different needs and preferences. Future research should investigate their perspectives on employment, education, and training services. In addition to the structure of the DCE survey, along with following a rigorous process in the development of the attributes and levels, there could have been some youth service priorities not assessed. Furthermore, as some of the attributes and levels built on each other, these commonalities could have influenced preference elicitation for specific service attributes. We tried to ensure that the survey was youth-friendly for all youth, however due to the cognitive capacity required to complete the survey, some youth with greater mental health and learning challenges may have been missed.

## Conclusions

This study identified employment, education, and training service preferences among Upcoming youth and those at-risk of Upcoming status in Canada. The findings indicate a need at the federal, provincial/territorial, and local level to create a service model that supports school and job opportunities long-term; mental health and wellness; and building holistic skills. The model also requires community-based and youth-centred approaches in the design and delivery of these services. Our findings further support the need for widespread policy support for broader-spectrum IYS for Upcoming youth and those at-risk of Upcoming status.

### Supplementary Information

Supplementary Material 1. Final List of Discrete Choice Experiment Attributes and Levels.

## Data Availability

No datasets were generated or analysed during the current study.

## Abbreviations

ABIC: Akaike’s Bayesian Information Criterion
AIC: Akaike Information Criterion
BIC: Bayesian Information Criteria
CAIC: Consistent Akaike Information Criterion
CAMH: Centre for Addiction and Mental Health
COVID-19: Coronavirus disease 2019
DCE: Discrete Choice Experiment
GAIN-SS: Global Appraisal of Individual Needs Short Screener
I: Importance Scores
IPS: Individual Placement and Support model
ISPOR: International Society for Pharmacoeconomics and Outcomes Research
IYS: Integrated Youth Services
MHSU: Mental health and substance use
NEET: Not in education, employment, or training
R: Rankings
SE: Standard Errors
YWHO: Youth Wellness Hubs Ontario
