# Supplementary material for: Youth not engaged in education, employment, or training: a discrete choice experiment of service preferences in Canada
Source: BMC Public Health. 2024 May 27;24:1402. doi: 10.1186/s12889-024-18877-0 (PMC11129463; doi:10.1186/s12889-024-18877-0)
Supplement: Supplementary file 1 — Supplementary Material 1. Final List of Discrete Choice Experiment Attributes and Levels. [file 12889_2024_18877_MOESM1_ESM.docx]

**Additional File 1.** Final List of Discrete Choice Experiment Attributes and Levels

| **Attribute** | **Level** |
| --- | --- |
| Mentorship | 1. Provide a mentor with work experience in the field I am interested in. |
|  | 1. Provide a mentor who has a similar background to me but may not be from my field of interest. |
|  | 1. Provide a peer mentor who recently went through “Services for Success: EET” program, but may not be from my background or field. |
| Skills for School and Job Success | 1. Free training focused on concrete skills like time management. |
|  | 1. Free training that includes concrete skills like time management AND problem solving. |
|  | 1. Free training that includes concrete skills like time management and problem solving AND soft skills like professionalism, communication and relationship building. |
| Technical Skills | 1. Provide a web-based resource of training courses for technical skills (e.g., computer, construction, hospitality, social media design skills) to show me what’s available in the community or online. |
|  | 1. Provide support to find and access training programs for technical skills (e.g., computer, construction, hospitality, social media design skills) in the community or online. |
|  | 1. Provide direct access to training programs for developing technical skills (e.g., computer, construction, hospitality, social media design skills) within “Services for Success: EET” program. |
| Life Skills | 1. “Services for Success: EET” program should focus only on skills directly related to getting and keeping a job/school placement. |
|  | 1. “Services for Success: EET” program should integrate life skills like managing finances and taxes. |
|  | 1. “Services for Success: EET” program should integrate life skills like managing finances and taxes AND skills like self-care, cooking or car-care. |
| Basic Income | 1. Provide me with basic income while I’m in postsecondary school or training. |
|  | 1. Provide me with basic income until I’m 25 years of age, regardless of my school or job status. |
|  | 1. Provide me with basic income until I’ve secured employment that matches the basic income level. |
| Networking Opportunities | 1. Do not offer networking skills or opportunities as part of “Services for Success: EET” program. |
|  | 1. Teach me skills to network as part of “Services for Success: EET” program. |
|  | 1. Teach me skills to network and provide opportunities to network with people in my area of interest as part of “Services for Success: EET” program. |
| Securing a Work or Educational Placement | 1. Support to find temporary job positions or educational placements to explore career options and fit. |
|  | 1. Support to secure any job position or educational placement, without considering longer term career interests or paths. |
|  | 1. Support to secure long term job positions or educational placements that align with career interests and long term goals. |
| Career Counselling | 1. Provide career counselling to help me figure out my career goals. |
|  | 1. Provide career counselling to help me figure out my career goals and how to create a resume/CV. |
|  | 1. Do not provide me with career counselling. |
| Access to Free Mental Health and Substance Use Services | 1. Include access to self-directed online mental health resources to support job and school success. |
|  | 1. Include on-site group mental health services to support job and school success. |
|  | 1. Include on-site individual mental health services to support job and school success. |
| Support for Mental Health and Wellness in the Workplace | 1. Teach me how to advocate for myself in the workplace. |
|  | 1. Provide supports during job onboarding to understand my rights. |
|  | 1. Provide ongoing access to a support worker who can help me secure accommodations (e.g., flex hours, specialized equipment) for me in the workplace. |

EET: Education, Employment, and Training
